# Pathology of Elephantiasis

**Published:** 1847-11

**Authors:** 


*Pathology of Elephantiasis.*—M. Cazenave has now under his care, at the Hospital St. Louis, a case of elephantiasis affecting the entire right leg. The thigh is so enormously distended, that its circumference surpasses that of the woman's body suffering with it; but with this increased size there is no deformity. The leg, on the contrary, is not only enlarged, but deformed, presenting hard, projecting masses, extending down to the instep, and the foot is also large and deformed. According to M. Cazenave, this elephantiasis of the Arabs, consists in a pure and simple hypertrophy of the affected parts—hypertrophy peculiar so far as it is connected necessarily with inflammation of the lymphatics. Ordinarily, and such has been the history of the present case, the disease commences suddenly, by a sharp and deep-seated pain along the course of the lymphatic vessels, which are soon transformed into hard, knotty cords; the limb then becomes the seat of an erysipelatous inflammation, which disappears, but this only for a time, being again reproduced and continuing for a greater or less time. This state of things may go on for several years. The cellular tissue is consecutively inflamed, and a more or less considerable tumefaction is set up, which augments at every fresh outbreak of the disease. Although tolerably soft at first, and, as it were, œdematous, the swelling at length gets hard, and resists the impression of the fingers. It is then the knobs or lumps make their appearance, and which produce the characteristic deformity of elephantiasis, knobs which will extend onwards to the feet or hands, (according as the legs or arms are affected,) and are sometimes separated by deep circular fissures, the whole accompanied by the normal colour of the skin, or by a duller whitensss than is seen in the healthy state. The soft tissues alone are affected, unless the disease is of very long standing, and the deep fissures have degenerated into wounds and ulcers, which may bring on consecutive disease of the bones. It is very peculiar that elephantiasis is observed much more frequently in the lower extremities than in the upper. However, there was a case some years since under M. Sanson, at La Pitié, of a very bulky elephantiasis affecting a woman's right arm.

It is a difficult matter to determine the treatment to be adopted. In M. Cazenave's patient, compression has been tried by means of a wide bandage, with agaric placed under it, at places much tumefied. Internal medicines, as mostly happens, have proved quite ineffectual. Since the compression has been employed, certain parts of the limb have notably diminished in volume, although a perfect cure cannot be hoped for, on account of the old standing of the malady, and the enormous degree at which the swelling has arrived.—*London Lancet.*

